# Cognitive recovery of post critical care patients with and without COVID-19: differences and similarities, an observational study

**DOI:** 10.1186/s42466-024-00349-w

**Published:** 2024-10-22

**Authors:** Anna Gorsler, Christiana Franke, Anneke Quitschau, Nadine Külzow

**Affiliations:** 1 Clinic for Neurological Rehabilitation, Fachklinik Für Neurologische Frührehabilitation, Kliniken Beelitz GmbH, Paracelsusring 6a, Beelitz-Heilstätten, 14547 Brandenburg, Germany; 2 Faculty of Health Sciences Brandenburg, Brandenburg Medical School Theodor Fontane, Brandenburg, Germany; 3 Department of Neurology and Experimental Neurology, Charité-Universitätsmedizin Berlin, Berlin, Germany

**Keywords:** COVID-19, Cognitive impairment, PICS, Intensive care unit, Recovery, Rehabilitation

## Abstract

**Background:**

Coronavirus disease (COVID-19) patients treated in an intensive care unit (ICU) are at high risk of developing cognitive impairments of a “post-intensive care syndrome” (PICS). We explored whether critically ill COVID-19 and non-COVID-19 survivors differ in their post-ICU recovery course in terms of severity and affected cognitive domains.

**Methods:**

An observational prospective study was conducted in a German post-acute neurological early rehabilitation clinic. Critically ill patients with or without SARS-CoV-2 infection (at least mechanically ventilated for one week) underwent repeated standardized assessments during their subsequent inpatient rehabilitation stay. Cognitive functions (information processing speed, learning, recognition, short-term and working-memory, word fluency, flexibility) assigned to different domains (attention, memory, executive functions) were assessed as primary outcome. Secondary outcomes included mental (depression, anxiety) and physical (Barthel index, modified ranking scale) state.

**Results:**

Out of 92 eligible patients (screened between June 2021 and August 2023), 34 were examined, and 30 were available for analysis (15 per group). Both groups were ventilated for a similar period (COVID-19 vs. Non-COVID-19: median: 48 vs. 53 days). Patients of COVID-19 group spend on average 10 days longer at ICU and developed slightly more complications, but subsequent inpatient rehabilitation was of comparable duration (median: 36.5 vs. 37 days). On the group-level both groups showed similar cognitive dysfunctions with striking impairments (normative T-scores < 41) in information processing speed, word fluency, flexibility, and recognition memory on admission. Significant gains until discharge were only revealed for information processing speed in both groups (main effect visit, mean difference [95%CI] − 7.5 [− 13.1, − 2.0]). Physical and mental state were also similarly affected in both groups on admission, but improved over time, indicating that overall recovery for higher-order cognitive functions is slowest. Interestingly, majority of patients stated correctly being still physically disabled, while a discrepancy was found between subjective and objective evaluation of cognitive health.

**Conclusions:**

Results suggest a substantial overlap of cognitive, mental and physical dysfunction in post-acute recovery of ICU survivors independent of SARS-CoV-2 infection which warrants further monitoring to reduce the risk of long-term burden and enable a return to previous functionality.

**Trial registration:**

Retrospectively registered at https://drks.de/search/de/trial/DRKS00025523, 21.06.2021.

## Background

SARS-CoV-2 Virus is known to trigger a variety of neurological and neuropsychiatric symptoms [1]. Regardless of the individual severity of the acute Coronavirus disease 2019 (COVID-19), ongoing cognitive impairments, fatigue, anxiety and depression are reported as part of a post-COVID-19 condition [2]. Even one year after intensive care unit (ICU) treatment around 75% of COVID-19 ICU survivors still complain of physical limitations, 25% of emotional stress, and 15% of cognitive impairments [3].

Patients who require intensive care during the acute infection are at risk of developing clinical symptoms of a “post-intensive care syndrome” (PICS) [4]. PICS is characterized by new or increased impairment of cognitive, mental and/or physical functions that outlast the hospitalization. PICS is, by definition, present when one or more of these three domains are affected [5]. Cognitive deteriorations include attention, memory, executive functions and/or changes in behavior [6]. The impairments can occur immediately after the ICU stay or later [7], and represent a major burden for both, ICU survivors and the health and social care system since cognitive deficits in particular hamper reintegration into daily live. Critically ill COVID-19 patients are often faced with a prolonged hospital stay and possible deep sedation, and are of increased risk to develop serious PICS [8, 9]. However, little is known about post-ICU cognitive recovery in patients with and without SARS-CoV-2 infection and whether they recover differentially in terms of severity and affected cognitive domains. This would have implications on therapeutic needs of ICU survivors and timely management of their aftercare. This study systematically investigated the post-acute clinical course and outcome of prior ventilated patients requiring intensive care with and without SARS-CoV-2 infection primary with regard to cognitive outcomes, and secondary to mental and physical state between admission and discharge from early inpatient rehabilitation.

## Methods

### Study population

This prospective observational study (German trial register www.drks.de: DRKS00025523, retrospectively registered at 21.06.2021) was part of a collaboration between the neurological post-COVID outpatient clinic of Charité Universitätsmedizin Berlin and the Kliniken Beelitz GmbH Brandenburg (neurorehabilitation clinic), approved by the local Ethic Committee (State medical association Brandenburg; postCov-Cog-2021-2073-NIS ff), and carried out according to the declaration of Helsinki. Inpatients (18–90 years) at the Kliniken Beelitz who had previously received intensive care treatment because of COVID-19 or other diseases and mechanical ventilation (≥ 1 week) were included with prior written consent. Additional inclusion criteria were i) Group 1 (COVID-19): PCR confirmed SARS-CoV-2 infection along with a COVID-19 associated rehabilitation diagnosis (e.g., critical illness polyneuropathy or myopathy (CIP/CIM)), ii) Group 2 (Non-COVID-19): CIP/CIM patients with primary pulmonary disease or other non-cerebral caused diseases. Exclusion criteria comprised: condition after cardiopulmonary resuscitation, presence of acute or pre-existing structural brain damage, severe hepatic insufficiency (hepatic encephalopathy), severe cognitive or communicative deficits that affect capacity to consent, or proven SARS-CoV-2 infection in the Non-COVID-19 group.

### Procedure

**Fig. 1:**
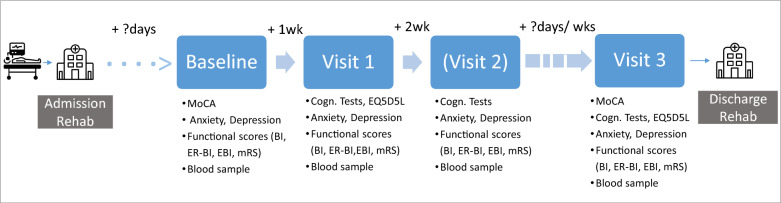
Schematic overview of the study: After admission of ICU survivors to the rehabilitation clinic patients underwent a baseline visit given that they were able to communicate beyond yes/no mode (with voice valve when necessary), and were attentive for at least 15 min. During the brief baseline visit global cognition (MoCA), mood (anxiety, depression), and functional scores (BI, ER-BI, EBI, mRS) were assessed and blood samples were taken (clinical routine). Visit 1 was scheduled one week after baseline and included cognitive tests to measure attention, memory and executive functions, and a questionaire concerning health-related quality of life (EQ5D5L) in addition to the repeated recording of mood, functional scores, and blood samples. All measurements (but without EQ5D5L) were re-assessed three weeks after baseline (visit 2), except discharge was at near future, then visit 2 was skipped and visit 3 was conducted. Visit 3 comprised the re-assessment of all visit 1 measurements and MoCA, and was always scheduled shortly before discharge. Therefore, number of visits and time lag/interval between visit 2 and 3 was variable between patients depending on length of stay. MoCA – Montreal cognitive Assessment, BI – Barthel Index, ER-BI – Early Rehabilitation Barthel Index, EBI – Extended Barthel Index, mRS – modified Rankin Scale, EQ5D5L – Euroqol 5 dimension 5 levels

Figure 1 summarizes the timeline and administered measurements. Number of visits and time between the last two visits varied depending on the individual length of stay. Once an eligible patient was able to communicate beyond “Yes/No-mode” and to maintain attention for at least 15 min, a brief baseline visit was scheduled. Baseline measurements included: global cognition (Montreal Cognitive Assessment (MoCA)) [10], depressive and anxiety symptoms (state and trait anxiety depression inventory (STADI), state version) [11], functional scores such as the modified Rankin Scale (mRS) [12], Barthel Index (BI) [13, 14]), early rehabilitation ER-BI [15], extended BI (EBI, measuring cognitive aspects of activities of daily living) [16], and inflammatory blood markers (C-reactive protein (CRP), leukocytes). One week after baseline (visit 1), assessments for attention, memory, executive functions (details below, and also Fig. 3A), and a self-assessment of health-related quality of life (EQ5D5L, https://euroqol.org/) [17, 18] were applied, and recording of mental parameters, functional scores, and inflammatory markers were repeated. All measurements of visit 1 (except EQ5D5L) were re-assessed three weeks after baseline (visit 2) unless patients were already scheduled for discharge. In this case, visit 2 was skipped and the final visit 3 (scheduled shortly before discharge) was conducted. Visit 3 comprised all measurements of visit 1 and the MoCA. Additionally, patients provided self-estimations about their physical and cognitive recovery. To avoid overburdening visits were kept rather short and, if necessary, scheduled on two days.

### Assessments

Widely accepted validated neuropsychological tests/ questionnaires (German versions) were applied to assess cognitive performances (attention, executive function, memory) as main outcomes. According to the known PICS domains secondary outcomes include the mental state (anxiety/ depression), and physical status (BI, mRS).

#### Cognitive assessments

The Trail Making Test (TMT) [19] measures the time to connect numbers 1–25 in numerical order (TMT-A: indicator for information processing speed, visual attention), and the time to alternately link the numbers 1–13 and letters A–L in ascending order (TMT-B: cognitive flexibility). The Regensburger word fluency test (RWT) [20] is associated with cognitive flexibility and requires to name as many words as possible that begin with a certain letter within one minute. The digit span (Wechsler Memory Scale revised) [21] comprises the repetition of a gradually increasing sequence of numbers in identical (forward) and reverse (backwards) order as a measure of short-term (STM) and working memory (WM) capacity. Memory function was tested by using word list learning. Different tests of varying difficulty depending on the current attentional capacities, premorbid condition, and level of education were used. The word list was either taken from the Nuernberger Age Inventory (NAI) [22], CERAD (Consortium to Establish a Registry for Alzheimer’s Disease test battery) [23], or the (most demanding) verbal learning and memory test (VLMT) [24]. All tests required at least learning and immediate free recall as well as a delayed recognition with learnt and new words. The list lengths varied between 8 (NAI) and 15 (VLMT) words to be learned. Global cognition (MoCA) scores range from 0 to 30.

#### Mental state

Mood (anxiety and depressive symptoms) was assessed with the STADI, which consists of 20 items that ask for the frequency of certain feelings (e.g., worry, tension) at this moment (state-form) on a Likert scale from 1 (almost never) to 4 (almost always). Points are added to an anxiety and depression score (higher scores indicate higher levels of anxiety/depression).

#### Physical status

BI and mRS were used as indicators. BI indicates independence in basic daily activities on a scale from total dependence (0) to total independence (100), and mRS describes physical disability from 0 (no symptoms) to 5 (severe disability).

Sociodemographic data, data obtained from routine hospital documentation, basic information about ICU stay (length, complications), comorbidities, duration of ventilation, information about medical devices and medication were additionally recorded. CRP and leukocyte levels were determined from routine blood samples, which were all analyzed by the same designated laboratory (Labor Potsdam, Germany).

### Data aggregation

To ease the comparison and interpretation of individual cognitive and mental performance we used standardized T-scores which indicate performance relative to the normative sample (T-scores ≥ 40 and ≤ 60 are considered normal). In some cases, it was necessary to convert the given value into a T-score: NAI: T = 5 * (C − 5) + 50; CERAD: T = 10 * z + 50 first. Whenever a raw value (RWT, TMT) was not assigned to a percentile rank in the norm table it was estimated by linear interpolation. Percentile ranks were then transformed into a T-score using the psychometrica-tool (https://www.psychometrica.de/normwertrechner.html [25]). Data on cognitive, mental and physical function were further subsumed to PICS domains in two steps:

1) Dichotomizing data according to ‘impaired’ and ‘normal’ (impaired refers to cognitive function T < 40, anxiety/depression T > 60, mRS > 2 and BI < 61).

2) If at least one measured symptom was outside the accepted range, the corresponding higher-level PICS domain was considered affected.

Missing values occurred for individual tests because of time constraints, patient refusal or inability to perform a particular task (e.g. hand dysfunction) resulting in varying sample sizes. All data (raw- and standardized) were entered and managed in a secure web-based software REDCap (Research Electronic Data Capture) [26].

### Statistical analysis

Given the observational and explorative nature of this study no prior sample size calculation was done. To characterize groups descriptive analysis including count and percentage, median and interquartile [IQR], or mean and standard deviation (SD) were applied. If appropriate, groups were further compared by non-parametric Mann–Whitney u-test (reported with Hodges-Lehman-median difference and 95%CI), or χ^2^-tests for categorical variables or counts. Within- and between-subject associations on primary (cognitive) and secondary outcomes were investigated by separate linear mixed models (random intercept). The models included group (COVID-19, Non-COVID-19 (reference)) as fixed factor, repeated measurements (visits 3 = reference) as level-one units nested in individuals (level-two units), and a group by visit interaction term to model different recovery courses between the groups. Statistical analyses were performed on T-scores for cognitive and mental data. Analyses were conducted by SPSS (IBM Version 29) within an explorational framework (not corrected for multiple testing).

## Results

**Fig. 2:**
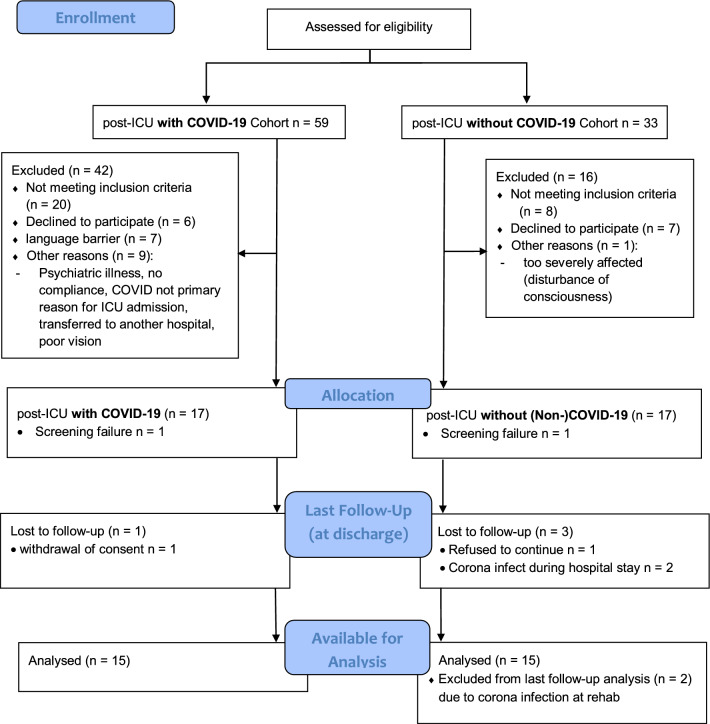
Flow chart of the study. Ninty-two eligible patients were enrolled, of whom 58 patients did not meet the inclusion criteria or declined, leaving a total of 34 consenting patients. Seventeen patients each could be allocated to the COVID-19 and non-COVID-19 group. In the COVID-19 group, two patients had to be excluded, one due to a history of stroke (screening failure) and one due to withdrawal of consent. In the Non-COVID-19 group two patients dropped out: one refused further participation and in one patient a previous mild COVID-19 infection became known only after inclusion (screening failure). During the rehabilitation stay two patients of the Non-COVID-19 group became infected with COVID-19 and had to be excluded from the last follow-up measurement

Out of 92 eligible patients (COVID-19: 59, Non-COVID-19: 33; between June 2021 and August 2023), 58 patients were initially excluded (n = 28 failed inclusion criteria, n = 13 declined, n = 7 insufficient knowledge of German, n = 10 for other reasons, see Fig. 2), and four during the course of the study (n = 2 screening failure, n = 1 withdrawal, n = 1 refused) leaving 15 patients per group for baseline analysis. Two patients were lost to follow-up (Non-COVID-19) due to a SARS-CoV-2 infection during their inpatient stay.

Both groups were similar in terms of age, gender ratio (more males than females) and self-care in daily living (Table 1). More than half of the patients were already retired, one was unable to work (COVID-19: 53%, Non-COVID-19: 60%) before hospitalization. In the COVID-19 group the majority was un- or incompletely vaccinated (67%), whereas in the Non-COVID-19 group almost all patients were fully vaccinated (86%). Patients of both groups suffered in median from three pre-existing diseases, the most common being hypertension. Distribution of comorbidities was similar between groups, with the exception of Chronic Obstructive Pulmonary Disease (COPD)/ asthma and heart failure, which occurred more frequently in the Non-COVID-19 group. This is probably due to the recruitment strategy for control patients.

**Table 1:** Demographic and clinical characteristics of cohorts, and main results of test statistics

	Total	COVID-19	Non-COVID-19	z or χ2	*p*
N	30	15	15		
Age in years, median [IQR]	64 [57, 68]	61 [54, 67]	64 [57, 69]	− 1.02^a^	0.31
Male, female (n)	20, 10	11, 4	9, 6	0.60^b^	0.44
Children, no children (n)	27, 3	13, 2	14, 1	2.35^b^	0.67
*Premorbid status* N	30	15	15		
Highest educational degree: vocational education, university degree (n)	21, 9	9, 6	12, 3	1.53^b^	0.47
Number of educational years (max.21), median [IQR]	13 [12, 16]	14 [12, 16]	13 [12, 15]	− 1.44^a^	0.15
Retired, incapable of work, employed or seeking a job (n)	16, 1, 13	7, 1, 7	9, 0, 6	7.39^b^	0.19
Existing, no existing care grade (n)	2, 28	0, 15	2, 13	2.14^b^	0.34
**Vaccination status (n): complete, incomplete, no**	16, 3, 9	**4, 2,8**	**12, 1,1**^**c**^	9.78^b^	.01
*Comorbidities* N	30	15	15		
No: n/ yes: median [IQR]	2/3[2, 5]	2/3 [1, 3]	0/3 [2, 5]	− 1.33^a^	.18
Diabetes (n)	11	6	5		
Hypertension (n)	20	9	11		
Renal insufficiency (n)	2	1	1		
**COPD/Asthma (n)**	5	**0**	**5**		
Atrial fibrillation (n)	5	3	2		
**Heart failure (n)**	7	**1**	**6**		
Obesity (n)	9	5	4		
CHD (n)	5	1	4		
History of myocardial infarction (n)	2	0	2		
Autoimmune disease (n)	2	2	0		
History of or current haemato-oncological (n)	2	1	1		
Anxiety, depression (n)	0	0	0		
Other pre-existing chronical disease (n)	5	2	3		
Other pre-existing illnesses (n)	18	8	10		
*Acute clinic* N	30	15	15		
**Days in ICU: median [IQR]**	29 [23.5, 49]	**37 [25, 56]**	**25 (20, 34]**	− 2.0^a^	0.05
**complications: no (n)/ yes median [IQR]**	1/3 [2, 4]	**1/4 [3, 5]**	**0/2 [2, 3]**	− 3.12^a^	0.002
Left ventricular assist device (n)	0	0	0		
**ECMO (n)**	7	**6**	**1**		
**Thrombosis (n)**	5	**5**	**0**		
Pulmonary Embolism (n)	3	3	0		
Myocardial infarction (n)	1	0	1		
Dialysis (n)	7	4	3		
Delirium (n)	20	12	8		
**ARDS (n)**	9	**8**	**1**		
Epileptic seizure (n)	3	3	0		
Sepsis (n)	19	9	10		
Liver failure (n)	1	1	0		
Anxiety, depression (n)	4	2	2		
**Multidrug-resistant pathogens (n)**	4	**4**	**0**		
*ADMISSION TO REHAB* N	30	15	15		
Days ventilated (median [IQR])	52 [33, 68.5]	48 [23, 80]	53 [48, 67]	− 0.15^a^	0.885
Days between ICU discharge and rehab: median [IQR]	0 [0, 8]	0 [0, 11]	0 [0, 2]	− 0.72^a^	0.47
*Rehab phase*				3.43^b^	0.18
Phase B with ventilation (n)	21	9	12		
Phase B without ventilation (n)	6	3	3		
Phase C (n)	3	3	0		
**Barthel Index: median [IQR]**	5 [0, 10]	**5 [0, 45]**	**0 [0, 5]**	− **2.04**^a^	**0.041**
Early rehab Barthel Index: median [IQR]	− 200 [− 200, − 100]	− 150 [− 200, 0]	− 200 [− 200, − 100]	− 1.14^a^	0.25
*Neurological examination*					
Hemiparesis (n)	0	0	0		
Tetraparesis (n)	27	13	14		
Delirium (n)	6	1	5		
**Dysphagia (n)**	21	**8**	**13**		
Dysarthria (n)	1	1	0		
*Medical devices*					
Nasogastric feeding tube (n)	14	7	7		
PEG (n)	4	1	3		
Tracheostomy (n)	22	10	12		
Invasive ventilation > 6 h/d (n)	19	8	11		
Noninvasive ventilation (n)	1	0	1		
Urinary catheter (n)	25	11	14		
No devices (n)	5	4	1		
*Medication*					
NOAC (n)	2	2	0		
LMWH (n)	26	12	14		
Vitamin K antagonists (n)	1	1	0		
Antidepressant (n)	5	3	2		
Sedatives (n)	10	4	6		
Neuroleptics (n)	11	5	6		
Antiepileptics (n)	5	3	2		
Anticholinergics (n)	2	0	2		
Antihypertensives (n)	28	14	14		

Data is given as absolut frequencies, or median [Interquartile range IQR: 25th and 75th percentile], COPD-Chronic obstructive pulmonary disease; CHD-Coronary Heart Disease; ECMO-Extracorporeal membrane oxygenation; ARDS-Acute Respiratory Distress Syndrome; ICU-intensive care unit; PEG-percutaneous endoscopic gastrostomy; NOAC-new oral anticoagulant; LMWH-low molecular weight heparin; known risk factors of PICS are shown with a grey background; most apparent differences are printed in bold; ^a^z values of Wilxocon-Mann–Whitney-Test; ^b^χ^2^ values of Chi-square test; *p* values (not corrected for multiple testing); ^c^ no information for one patient available

### ICU stay

Compared to controls, COVID-19 patients were treated longer at ICU (Hodges-Lehmann-median-difference [95%CI]: 10 [0, 26]) and developed slightly more complications (Hogges-Lehmann-median-difference [95%CI]: 2 [1, 3]). Especially, severe acute respiratory distress syndrome (ARDS) occurred more often (COVID-19, Non-COVID-19: 53% vs. 7%) resulting in more frequent use of extracorporeal membrane oxygenation (ECMO) among COVID-19 survivors. Further, thrombosis and infections with multidrug resistant pathogens were more common in COVID-19 (27%) than in Non-COVID-19 (0%) patients. Beyond that, delirium (67%) and sepsis (63%) were the most frequently reported complications in both groups (Table 1).

### Admission to rehab

Duration of mechanical ventilation was similar between groups (Hodges-Lehmann-median-difference [95%CI]: − 3 [− 23, 25]). A high proportion of patients was still mechanically ventilated on admission (70%) and prolonged weaning via tracheostomy had to be continued in the early rehabilitation center. Functional dependency was high, and somewhat higher in the Non-COVID-19 group. Ninety percent of patients suffered from acquired neuromuscular weakness (tetraparesis), 53% of COVID-19 and 87% of Non-COVID-19 group were diagnosed with dysphagia, and most patients (83%) were admitted with a urinary catheter. Almost all patients were treated with antihypertensive drugs (93%) and anticoagulation (97%), about one third received sedatives and neuroleptics (Table 1).

### Cognition (primary outcome)

**Fig. 3:**
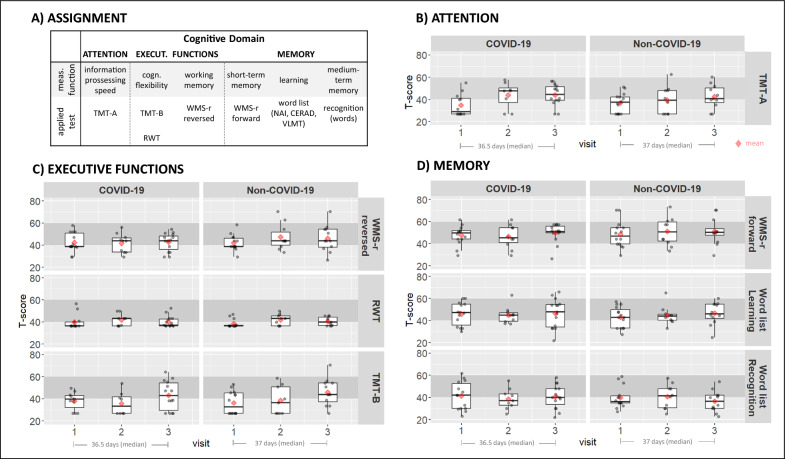
Description of cognitive domains and test results. **A** Assignment of the applied cognitive test to a measured function and the associated superordinate cognitive domain. **B**–**D** Boxplots of all cognitive measures during the inpatient stay at visit 1, visit 2, and visit 3 seperated by groups (COVID-19-left, Non-COVID-19-right). Data are given as T-scores. The underlying grey shadded box marks the area of accepted performance range (T-scores between 40 and 60). The superimposed red diamond represents the mean of respective group. The plots are organized according to the assigned cognitive domains and show at the group level an increase in attentional scores (TMT-A) in both groups (B). With regard to executive functions, slight improvements in working memory (WMS-r reversed)), but ongoing problems in cognitive flexibility (RWT, TMT-B) were observed in both groups (C). Within the memory domain continued problems were found in word recognition, but not learning or short-term memory (WMS-r forward) in both groups (D). Note, at visit 2 N is reduced in both groups, because in a number of patients vist 2 was skipped due to individual length of stay and visit 3 was already conducted. Meas.-Measurement, Execut.-Executive, Cogn.-cognitive, TMT-Trail Making Test, RWT-Regensburger Word Fluency Test, WMS-r-Wechsler Memory scale revised, NAI-Nuernberger Age Inventory, CERAD-Consortium to Establish a Registry for Alzheimer’s Disease, VLMT-Verbal Learning and Memory Test

The time span between visit 1 and visit 3 did not differ between groups (median in days [IQR]: COVID-19: 36.5 [10.5, 56.0], Non-COVID-19: 37.0 [15.5, 60]). Analysis of cognitive outcomes (Fig. 3A) revealed no substantial group differences for any of the parameters (details are reported in Table 2, and illustrated in Fig. 3B–D).

**Table 2:** Main results of linear mixed model analyses (factor group and visits) for different cognitive outcomes

Outcome			Memory									Executive function									Attention	
			STM (DS-for.)			Learning (words)			Recognition			Flexibility (TMT-B)			Word fluency (RWT)			WM (DS-back)			TMT-A	
			Mean diff. (SEM)	95%CI		Mean diff. (SEM)	95%CI		Mean diff. (SEM)	95%CI		Mean diff. (SEM)	95%CI		Mean diff. (SEM)	95%CI		Mean diff. (SEM)	95%CI		Mean diff. (SEM)	95%CI
Data pts tot		75			76			74			66			72			75			70		
Group (n-Cov = 0)			− 2.3 (2.5)	− 7.4, 2.7		1.0 (2.3)	− 3.5, 5.6		1.1 (2.5)	− 3.9, 6.1		− 0.1 (3.0)	− 6.1, 5.9		0.2 (1.2)	− 2.2, 2.7		− 2.8 (2.2)	− 7.2, 1.7		1.3 (2.7)	− 4.1, 6.8
Visit (visit 3 = 0)			− 2.5 (2.8)	− 8.1, 3.1		− 2.1 (2.9)	− 8.0, 3.8		2.0 (2.9)	− 3.8, 7.8		− 5.5 (3.4)	− 12.4, 1.5		− 1.3 (1.3)	− 4.0, 1.4		− 2.2 (2.7)	− 7.1, 2.8		− 7.5 (2.8)	− 13.1, − 2.0
Group x visit		N			N			N			N			N			N			N		
COV—n-COV	Visit 1	28	− 1.0 (4.0)	− 9.2, 7.1	29	2.9 (3.7)	− 4.7, 10.6	28	1.5 (4.3)	− 7.3, 10.4	25	1.7 (3.8)	− 6.3, 9.6	28	1.5 (2.0)	− 2.6, 5.5	28	0.3 (3.0)	− 5.9, 6.4	27	− 2.0 (3.6)	− 9.5, 5.5
	Visit 2	20	− 4.6 (5.2)	− 15.5, 6.3	20	0.3 (3.6)	− 7.3, 7.9	19	− 2.1 (4.7)	− 12.0, 7.9	17	− 2.8 (5.8)	− 15.1, 9.5	18	− 0.2 (2.5)	− 5.5, 2.6	20	− 5.4 (4.6)	− 15.1, 4.3	18	4.6 (5.9)	− 7.8, 17.0
	Visit 3	27	− 1.4 (3.9)	− 9.4, 6.6	27	− 0.02 (4.6)	− 9.4, 9.4	27	3.9 (3.9)	− 4.1, 11.9	24	0.9 (5.7)	− 11.0, 12.7	26	− 0.5 (1.8)	− 4.3, 3.2	27	− 3.1 (3.9)	− 11.1, 4.9	25	1.5 (4.2)	− 7.2, 10.1

COV-COVID-19; n-COV-Non-COVID-19; STM-short-term memory; DS-for-digit span forward; DS-back-digit span backwards; WM-working memory; TMT-Trail Making Test; RWT-Regensburger Word Fluency Test; pts.-points; tot.-total; Ref.-Reference; diff-difference; SEM-standard error of the mean; CI-confidence interval; in bold significant effect

#### Attention

At the group-level, the time for completing the TMT-A indicates a slowed information processing for both groups at visit 1 (mean T-score (SD): COVID-19: 34.9 (10.2), Non-COVID-19: 36.9 (8.7)). The processing speed increased considerably over time (main effect visit) for both groups (visit 1–visit 3: mean difference [95%CI]: − 7.5 [− 13.1, − 2.0]) but was still in the lower normal range at visit 3 (mean T-score (SD): COVID-19: 44.2 (9.9), Non-COVID-19: 42.7 (10.9); Fig. 3B).

#### Executive function

On average, initial performance on TMT-B and word fluency tests (aspects of cognitive flexibility) were poor in both groups. On a descriptive level mean TMT-B performance improved over time (mean T-Score (SD) visit 1 vs. visit 3: COVID-19: 38.0 (2.4) vs. 43.0 (3.5); mean difference [95%CI]: − 5.1 [− 14.5, 4.3]; Non-COVID-19: 36.3 (2.8) vs. 42.2 (4.6); mean difference [95%CI]: 5.9 [− 16.1, 4.3]), but hardly any changes were observed in word fluency performance (mean T-score (SD) visit 1 vs. visit 3: COVID-19: 40.5 (2.1) vs. 40.0 (1.4); Non-COVID-19: 38.9 (1.0) vs. 40.2 (1.2)). Mean working memory performance was within the normal range at visit 1 and also at visit 3 (all mean T-Scores > 42, Fig. 3C).

#### Memory

Low and below-normal performance was observed on average in recognition tests at visit 1 (mean T-score (SD): COVID-19: 41.4 (3.0), Non-COVID-19: 39.8 (3.0)), while mean learning performance and STM capacity were within accepted limits (all mean T-scores > 42). For all three memory parameters (learning, recognition, STM capacity) performance remained at a similar level with no major improvements over time (Fig. 3D).

### Mental and physical state

At baseline increased anxiety levels (T-score > 62) were evident in both groups, while mean depression scores were elevated, but still within the accepted range (both mean T-scores < 60). Mental scores normalized over time (main effect visit (baseline-visit 3) mean difference [95% CI] for anxiety: 9.1 [3.3, 15.0], for depression: 14.9 [6.9, 22.9]; Table 3). BI index was higher (main effect visit (baseline-visit 3): mean difference [95%CI]: − 41.8 [− 51.8, − 31.7]) and mRS-scores lower (Wilcoxon tests: Hodges-Lehmann-median-difference [95%CI]: COVID-19: − 2.0 [− 2.5, − 1.0]), Non-COVID-19: − 1.5 [− 2.0, − 1.0]) in both groups at visit 3 compared to baseline indicating less functional dependency and disability on discharge (Table 3).

**Table 3:** Descriptive statistics of secondary outcomes, global cognition, health-related quality of life, and subjective health estimation

		N	Total	COVID-19	Non-COVID-19	Percentage	
				(Cov)	(n-Cov)	COV	n-COV
*Mood*		COV,n-COV	Mean (SD)	Mean (SD)	Mean (SD)	Above normal range (abnormal) in %	
Anxiety	Baseline	14, 13	62.4 (10.3)	62.4 (11.0)	62.4 (10.0)	71.4	53.8
	Visit 1	14, 15	59.4 (9.7)	60.8 (11.2)	58.1 (8.2)	57.1	40.0
	Visit 2	10, 9	55.8 (9.9)	53.9 (11.1)	57.9 (8.4)	30.0	33.3
	Visit 3	15, 11	53.0 (10.5)	51.5 (11.1)	55.0 (9.7)	13.3	36.4
Depression	Baseline	14, 13	58.2 (14.8)	59.3 (14.6)	56.9 (15.5)	42.9	46.2
	Visit 1	14, 15	54.8 (12.8)	53.9 (11.9)	55.6 (13.9)	35.7	53.3
	Visit 2	10, 9	53.4 (13.2)	48.6 (12.9)	58.8 (11.9)	30.0	55.6
	Visit 3	15, 11	43.2 (13.4)	43.0 (13.6)	43.5 (13.7)	13.3	18.2
*Functional scores*		COV,n-COV	Median [IQR]	Median [IQR]	Median [IQR]	Below cut-off in %	
BI	Baseline	15, 15	15 [5, 31.3]	15 [5, 45]	20 [10, 25]	93.3	100
	Visit 1	14, 15	27.5 [15, 45]	35 [15, 48.8]	20 [15, 40]	85.7	93.3
	Visit 2	10, 1	50 [40, 58.8]	55 [20, 61.3]	45 [40, 55]	80.0	100
	Visit 3	15, 13	65 [60, 72.5]	65 [60, 75]	65 [57.5, 67.5]	40.0	30.8
ER-BI	Baseline	15, 15	− 150 [− 150, − 87.5]	− 150 [− 150, 0]	− 100 [− 150, − 100]	–	–
	Visit 1	14, 15	− 100 [− 150, − 37.5]	− 100 [− 150,0]	− 100 [− 150, − 100]	–	–
	Visit 2	10, 1	− 50 [− 100, 0]	0 [− 112.5, 0]	− 50 [− 62.5, 0]	–	–
	Visit 3	15, 13	0 [− 50, 0]	0 [− 50, 0]	0 [− 50, 0]	–	–
EBI	Baseline	15, 12	85 [75, 90]	85 [80, 90]	75 [70, 90]	–	–
	Visit 1	14, 14	85 [75, 90]	85 [83.8, 90]	77.5 [73.8, 90]	–	–
	Visit 2	10, 1	90 [80, 90]	90 [80, 90]	85 [75, 90]	–	–
	Visit 3	15, 13	90 [80, 90]	90 [85, 90]	90 [75, 90]	–	–
mRS	Baseline	15, 15	4 [4, 5]	4 [3, 5]	4 [4, 5]	86.7	93.3
	Visit 1	14, 15	4 [3, 4]	4 [2.8, 4]	4 [4]	78.6	100.0
	Visit 2	10, 1	4 [3, 4]	3.5 [3, 4]	4 [3, 4]	90.0	90.0
	Visit 3	15, 13	2 [1.3, 3]	2 [1, 3]	3 [2, 3.5]	40.0	53.9
*Other*		COV,n-COV	Mean (SD)	Mean (SD)	Mean (SD)	Below cut-off in %	
MoCA*	Baseline	13, 14	22.7 (3.5)	23.5 (3.8)	21.9 (3.2)	69.2	85.7
	Visit 3	13, 9	24.9 (2.6)	25.6 (2.3)	23.9 (2.8)	53.8	77.8
EQ5D5L						–	–
*EQ index*	visit 1	14, 14	0.6 (0.3)	0.6 (0.3)	0.6 (0.4)	–	–
	visit 3	15, 10	0.8 (0.2)	0.8 (0.2)	0.8 (0.1)	–	–
*VAS-score*	visit 1	14, 14	50.1 (21.0)	47.2 (20.0)	52.9 (22.2)	–	–
	visit 3	15, 10	67.4 (17.1)	67.2 (18.5)	67.8 (15.6)	–	–
How many % regained (visit 3)						Subjectively fully (100%) recovered in %	
Of physical health		13, 9	74 [60, 80]	68 [55, 77.5]	80 [67.5, 80]	0	0
Of cognitive health		13, 9	99 [83.8, 100]	98 [82.5, 100]	100 [80, 100]	46.2	55.6

BI-Barthel Index; ER BI-Early Rehabilitation Barthel Index; mRS-Modified Rankin Scale; EBI-Extended Barthel Index; MoCA-Montreal Cognitive Assesment; EQ5D5L-European Quality of Life 5 Dimensions 5 Level (EQ index: 0-“very bad” to 1-“best possible state of health”); VAS-Visual Analogue Scale (0-“worst state of health” to 100-“best possible state of health”); *3 patients were not able to perform the drawing task of MoCA and were excluded from statistics

**Fig. 4:**
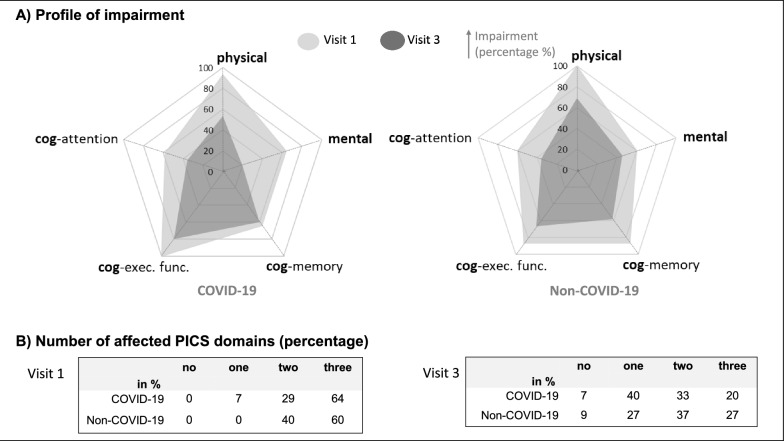
**A** Radar diagram to illustrate percentage of patients (axes: 0-100) with problems in the respective higher-level PICS-relevant domains (radii). Visit 1 (light grey) and visit 3 (dark grey) are superimposed to visualize changes over time across domains, seperately for the COVID-19 (left) and Non-COVID-19 group (right). Profiles are similar for both groups. At visit 1, colored in light grey, a high proportion (> 50%) of patients suffered from deficits in all domains. Over time, at visit 3, colored in dark grey, percentage decreased in both groups. On a descriptive level, the decline was similar for all domains in the Non-COVID-19, while in the COVID-19 group the strongest decline was observed within the mental domain. **B** Number of affected PICS domains for visit 1 (left) and visit 3 (right). The columns define if none, one, two, or all three of the higher-level PICS domains (physical, cognitve, mental) are impaired. The cells represent the number of patients (in percent) within the respective category (number of affected domains) separately for COVID-19 and Non-COVID-19 group

Overall, the majority (> 60%) suffer from overlapping problems in all PICS-domains (Fig. 4). Over time, the number of affected domains decreased: COVID-19 survivors showed most frequently one affected domain (cognitive), Non-COVID-19 survivors two (cognitive, physical). Only one patient per group demonstrated no impairments on discharge.

### Other parameters

As depicted in Table 3 both groups showed similar improvements (main effect visit: visit 1–visit 3) in self-reported health-related quality of life measured with the derived EQ index (mean difference [95%CI]: − 0.2 [− 0.3, − 0.02]) and the visual analog scale (VAS-score as indicator for general subjective health status: mean difference [95%CI]: − 17.2 [− 28.2, − 6.9]) of the EuroQol-questionnaire. Global cognition also increased over time (MoCA: baseline-visit 3: − 2.3 [− 4.2, − 0.5]). However, on discharge a notable percentage (> 50%) demonstrated mild cognitive impairment (MoCA < 26, [10]). None of the patients felt fully recovered regarding their physical health on discharge opposed to their cognitive health. Here, even 46.2% of COVID-19 and 55.6% of Non-COVID-19 patients stated that they had regained 100% of their previous condition. CRP-levels were elevated (> 5 mg/l) at baseline in COVID-19 (9/13 patients (69%)), and non-COVID-19 (9/15 patients (60%)) group, and partly on discharge (COVID-19: 5/15 (33%), Non-COVID-19: 8/12 (67%)). Leukocyte-levels were within the normal limits (male/female: ≥ 4.2/4.0 or ≤ 9.1/10.0 Gpt/l) in more than 65% at baseline (COVID-19: 10/13 patients, Non-COVID-19: 10/15 patients) and on discharge (COVID-19: 12/15 patients, Non-COVID-19: 8/12 patients).

### Situation on discharge

The majority of patients were discharged home (COVID-19: 78.6%, Non-COVID-19: 58.3%), two patients from the COVID-19 and three from the Non-COVID-19 group needed additional care at home. Three patients could not return to home: one (COVID-19) was admitted to an assisted living facility, two to an intensive care facility (Non-COVID-19). After rehabilitation, only 14% of patients needed a urinary catheter, one patient still required mechanical ventilation and one a gastric tube (both from the Non-COVID-19 group). Eighty-two percent of patients continued taking antihypertensive, but other pre-administered medications could be considerably reduced (sedatives: n = 0, neuroleptics: n = 3).

## Discussion

In this prospective study we compared the recovery process focusing on cognitive and other PICS-related domains (mental, physical) during early rehabilitation of COVID-19/ Non-COVID-19 ICU survivors. First, shortly after discharge from ICU, we found similar patterns of impairment in cognitive, mental and physical functions as well as no markedly different course of recovery during post-ICU inpatient early rehabilitation. Second, more than 85% of patients showed difficulties in at least one of the cognitive tests on discharge. Third, in both groups a mismatch between subjective perception and objective test performance was observed with regard to the evaluation of cognitive health.

On admission to early rehabilitation, in both groups median performance was impaired in all cognitive domains (attention, execution and memory [word recognition]). As information processing ability and speed (attention) is essential for higher-order cognitive functions, impairments can have a negative impact on executive or memory (recognition) functions [27]. However, despite information processing speed improved over time in both groups, performance in more complex tasks (word fluency, TMT-B, recognition memory) remained weak on a group-level during inpatient stay. This may indicate that higher-order cognitive functions recover more slowly and the achieved attentional level was probably still insufficient to promote executive functions or word recognition. Since executive functions like cognitive flexibility have been demonstrated as an important determinant for instrumental activities of daily livings [28], knowledge about deterioration on discharge is of great interests as it can impede return to independent daily living.

Despite the longer ICU stay in COVID-19 group, and contrary to expectations, the measured consequences and recovery of cognitive impairment and other PICS domains were similar in COVID-19 and Non-COVID-19 ICU survivors. Results agree with other recent reports [8, 29, 30]. It is not yet clear how the different pathologies and in particular the neurotropism of the SARS-CoV-2 virus, which is supposed to trigger cognitive disturbances [31], lead to the observed similarities. However, known risk factors such as delirium and sepsis [32] were evenly distributed between groups. ARDS, in contrast, occurred more frequently in the COVID-19 group (COVID-19, Non-COVID-19: 53% vs 7%), entailed further complications and prolonged ICU stay but without driving specific other cognitive dysfunctions compared to Non-COVID-19 critically ill patients. Thus, probably more general ICU-related factors contribute to cognitive difficulties [33]. Moreover, individual factors such as education, cognitive reserve, and social support may also play an important role in recovery [34].

Improvements were found in level of anxiety and depression, functional dependency and disability (BI, mRS). However, 13–36% of patients still showed increased anxiety/depression scores, and 31–54% suffered from noteable physical limitations on discharge. Taken together, almost all patients fulfill criteria of PICS on discharge as defined by at least one dysfunction in relevant PICS domains. In both groups the cognitive domain showed the slowest progress, specifically higher-order functions changed hardly during mid-term recovery. To conclude, we found substantial overlap in the post-ICU recovery regardless of pathogen or underling critical illness and add insights about post-acute changes in recovery to previous findings.

Finally, we would like to emphasize the discrepancy between subjective and objective cognitive health, in contrast to self-estimation of physical condition. Despite persisting cognitive deficits, the majority in both groups (COVID-19: 11/13, Non-COVID-19: 7/9 patients) stated that they have regained 80% or more of previous cognitive health. This overestimation might be explained by patient’s internal focus at survey (regaining functional independence after a life-threatening illness, regardless of the disease), and may not be comparable to ambulant non-ICU groups. Further, a protected, and cognitively less demanding inpatient setting may reduce awareness for cognitive problems. However, return to everyday life can then induce overburden, accompanied by increased depressive symptoms negatively affecting cognitive and physical functions, social participation and re-integration [35]. Thus, monitoring by objective assessments after discharge is highly recommended.

The strength of our study was the use of a prospective design and implementation of differentiated cognitive tests. However, some limitations must be acknowledged. Our study comprised only a small cohort size and a heterogeneous control group in terms of the underlying critical illness. Nevertheless, baseline characteristics and number of comorbidities were comparable between COVID-19 and Non-COVID-19 critical ill patients. A selection bias cannot be excluded because only ICU survivors suffering from CIP/CIM who were admitted to our neurological rehabilitation clinic were included. However, CIP/CIM affects a high proportion (50–80%) of intensive care patients [36]. Dependent on individual recovery number and time lag between visits varied between patients, but in the median the groups were similar.

## Conclusions

The main conclusion of this study is that COVID-19 and Non-COVID-19 critically ill patients do not differ in post-ICU early rehabilitation. A considerable proportion show cognitive dysfunction still on discharge. Cognitive complaints warrant further early monitoring and therapy for both groups even if the patients do not perceive themselves as largely cognitively compromised, in order to better prepare these vulnerable cohort to their return to daily demands and (working) routines.

## Data Availability

The data that support the findings of this study are available from the corresponding author, AG, upon reasonable request.

## Abbreviations

ARDS: Severe Acute Respiratory Distress Syndrome
BI: Barthel Index
CERAD: Consortium to Establish a Registry for Alzheimer’s Disease test battery
CI: Confidence Interval
CIM: Critical Illness Myopathy
CIP: Critical Illness Polyneuropathy
COPD: Chronic Obstructive Pulmonary Disease
COVID-19: Coronavirus disease
CRP: C-reactive protein
EBI: Extended Barthel Index
ECMO: Extracorporeal Membrane Oxygenation
EQ5D5L: Euro Qulaity of Life Measurement 5 Dimensions, 5 Levels
ER-BI: Early Rehabilitation Barthel Index
ICU: Intensive Care Unit
IQR: Interquartile Range
MoCA: Montreal Cognitive Assessment
mRS: modified Rankin Scale
NAI: Nuernberger Age Inventory
PCR: Polymerase Chain Reaction
PICS: Post Intensive Care Syndrome
REDCap: Research Electronic Data Capture
RWT: Regensburger Word fluency Test
SARS-CoV-2: Severe Acute Respiratory Syndrome Coronavirus type 2
SD: Standard Deviation
SPSS: Statistical Package für Social Sciences
STADI: State and Trait Anxiety Depression Inventory
STM: Short-term Memory
TMT: Trail Making Test
VLMT: Verbal Learning and Memory Test
WM: Working Memory
